# Shear Bond Strength of Polypropylene Fiber in Orthodontic Adhesive on Glazed Monolithic Zirconia

**DOI:** 10.3390/polym14214627

**Published:** 2022-10-31

**Authors:** Dhanabhol Riowruangsanggoon, Apiwat Riddhabhaya, Nattisa Niyomtham, Irin Sirisoontorn

**Affiliations:** 1 Department of Clinical Dentistry, Walailak University International College of Dentistry (WUICD), 87 Ranong 2 Road, Dusit, Bangkok 10300, Thailand; 2 Department of Oral Health Science, Walailak University International College of Dentistry (WUICD), 87 Ranong 2 Road, Dusit, Bangkok 10300, Thailand

**Keywords:** ceramic bracket, metal bracket, monolithic zirconia, precoated bracket, shear bond strength

## Abstract

This study aims to compare shear bond strength (SBS) and mode of failure (MOF) between ceramic and metal orthodontic brackets on glazed monolithic zirconia using non-woven polypropylene fiber adhesive. Sixty glazed and sintered zirconia blocks were divided into six groups and attached with orthodontic brackets as follows: CS, ceramic bracket with silane; CB, ceramic bracket with bonding agent; CBS, ceramic bracket with bonding agent and silane; MS, metal bracket with silane; MB, metal bracket with bonding agent; and MBS, metal bracket with bonding agent and silane. There was a statistically significant difference in mean SBS values (*p* < 0.001). The CS group showed the highest SBS value (23.42 ± 3.88 MPa). On the other hand, the lowest was found in the MB group, which was not statistically different from the CB group (3.26 ± 0.76 and 5.09 ± 1.50 MPa, respectively). The MS, MBS, and CBS groups showed no statistical difference compared to each other (15.57 ± 4.01, 13.23 ± 5.47, and 12.77 ± 4.43 MPa, respectively). SBS is highest when a ceramic bracket with silane is bonded to glazed monolithic zirconia.

## 1. Introduction

In the past, conventional ceramics were established to meet the needs of dentists and patients for superior aesthetic and natural-like restorations. Nevertheless, conventional dental ceramic restorations have mechanical limitations, such as low tensile strength, crack dissemination, fragility, fractural strength, marginal accuracy, wear resistance, and difficulty of reparation [1].

In the early 1990s, zirconia was developed for dentistry and was chosen as a substructure to help support more aesthetic materials. Zirconia is the hardest ceramic material used in dentistry and it also displays equal mechanical properties to those of some metals [2,3]. Zirconia has twice the flexural strength compared to conventional ceramics (900–1200 MPa). Moreover, high fractural strength (9–10 MPam^1/2^) has been demonstrated [2,4]. Therefore, of all the ceramics used in dentistry, zirconia is the hardest and strongest [5].

In the following years, the attention of researchers was given to the nanostructure modification of zirconia by adding yttrium oxide, called zirconia–yttria ceramics or tetragonal zirconia polycrystals (TZPs) [6]. TZPs were developed to meet the needs of the dental profession. By adding different amounts of yttrium oxide, the strength and translucency of nanocrystalline monolithic zirconia can be varied.

Monolithic zirconia crowns without layering ceramics have become increasingly popular as a result of CAD/CAM technology and increasing zirconia translucency [7,8]. Most of the time, monolithic zirconia is used on molar teeth to stop veneers from cracking [9].

Zirconia has evolved, but it still has significant drawbacks. To make zirconia look more natural, it must be glazed. The dental ceramics glazing procedure was developed to solve the issue. Glazing is formed by burning a thin, transparent silica glass coating on the surface or by firing the substructure up to the proper temperature for a few minutes to obtain a glossy ceramic surface [10]. Furthermore, glazed monolithic zirconia wears opposing teeth less than polished zirconia [11].

The number of adult orthodontic patients has recently increased. A comparatively higher percentage of people (about 45 percent) in the middle-age group are interested in orthodontic treatment [12]. Women between the ages of 25 and 35 comprise the majority of the adult orthodontic patient population [13]. Adult orthodontic patients typically lack a complete set of teeth when they begin treatment. In some cases, other diseases or even restorations such as fillings, crowns, or even bridges may be present.

**Figure 1:**
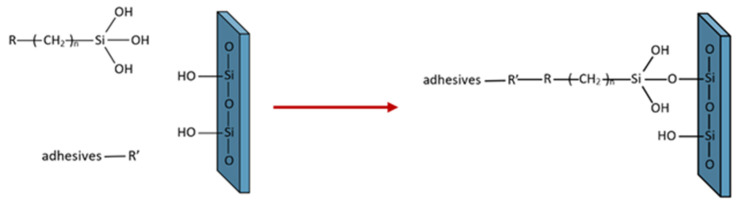
Illustration of a silane coupling agent forming a chemical bond between inorganic and organic substrates.

The currently present silane coupling agents in dentistry use tri-alkoxysilane, such as 3–methacryloxyproyltrimethoxysilane (MPS), as the reactive vital ingredient. The concentration of silane can vary between 1–10 *v/v*% in different marketable brands. Recent commercial resin composites in dentistry, a gold standard, contain the following components: resin matrixes, free-radical initiators and inhibitors or stabilizers, dyes (pigments), fillers, and silane coupling agents. The combination of the filler elements increases the mechanical properties and decreases the polymerization shrinkage after curing them [14]. Adding a silane coupling agent can reinforce the adhesion between the resin matrix and filler particles. During light curing, the functional group of the unhardened resin monomers reacts with the organofunctional group of the silane coupling agent to produce a chemical bond, such as –C–C–, as shown in Figure 1 [14].

**Figure 2:**
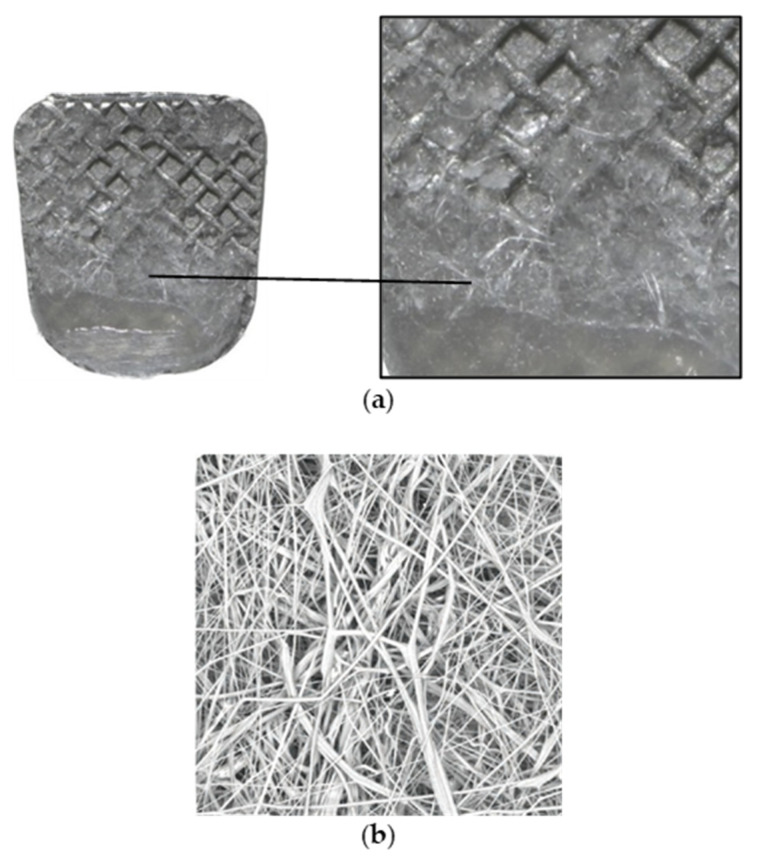
(**a**) Images of APC^TM^ Flash-Free adhesive remnant from 10× magnification light microscope digital camera. (**b**) The non-woven polypropylene fibers used in the APC^TM^ Flash-Free adhesive consist of randomly oriented, entangled fibers (3M^TM^ APC^TM^ Flash-Free Adhesive, Technical Overview).

A non-woven polypropylene fiber orthodontic adhesive was introduced using a novel material manufacturing technology (Figure 2). It was supplied, along with the bracket, in individually sealed blister packs to prevent the risk of cross-contamination. Moreover, this new technology claims to be comparable with the conventional adhesive in the aspect of shear bond strength in normal conditions or even after a load of an ultrasonic scaler was used to test it [15,16]. Another benefit is the reduction in chair time by eliminating the process of excessive flash removal on bonding procedures.

Nowadays, there are several types of orthodontic appliances. Ceramic brackets, lingual brackets, and even clear aligners have been developed to meet the aesthetic needs of adult orthodontic patients. Fixed orthodontic braces with aesthetically pleasing brackets have been reported to be among the most commonly used in adult patients [17]. Due to higher aesthetic requirements and the increasing use of monolithic zirconia, there is a potential to encounter patients who have both aesthetic needs and zirconia restorations in their mouths.

A systematic review of orthodontic bonding to porcelain in 2013 concluded that etching with 9.6% hydrofluoric acid for 1 min, rinsing for 30 s, air-drying, and then applying a silane coupling agent is the best protocol for bonding to porcelain. Regarding the dangerous effects of hydrofluoric acid etching, another alternative is sandblasting to create physical roughness, followed by silane application [18].

With fixed orthodontic brackets, the bond strength between teeth and appliances should exceed the force that is needed to move teeth successfully. Reynolds and colleagues stated that a maximum value of 5.9–7.9 MPa would be reasonable enough [19]. For non-glazed or polished monolithic zirconia, studies of bonding properties on metal and porcelain brackets found that proper shear bond strength can be provided for both types of brackets [20,21]. Moreover, the comparison between metal and porcelain brackets found that metal brackets provided more shear bond strength than porcelain brackets on polished monolithic zirconia [22]. The study regarding bonding properties on glazed monolithic zirconia so far, however, is still only on metal brackets, and the results also show excessive (>13 MPa) shear bond strength [23]. This study aims to compare shear bond strength and mode of failure between ceramic and metal orthodontic brackets on glazed monolithic zirconia using precoated, non-woven polypropylene fiber orthodontic adhesive for the advancement of polymeric materials in orthodontic clinical application.

## 2. Materials and Methods

### 2.1. Sample Preparation

**Figure 3:**
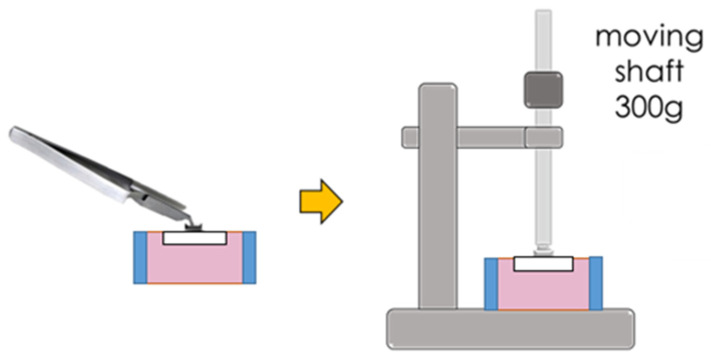
Precoated brackets were placed using moving shaft of 300 g after sample surface preparation.

**Figure 4:**
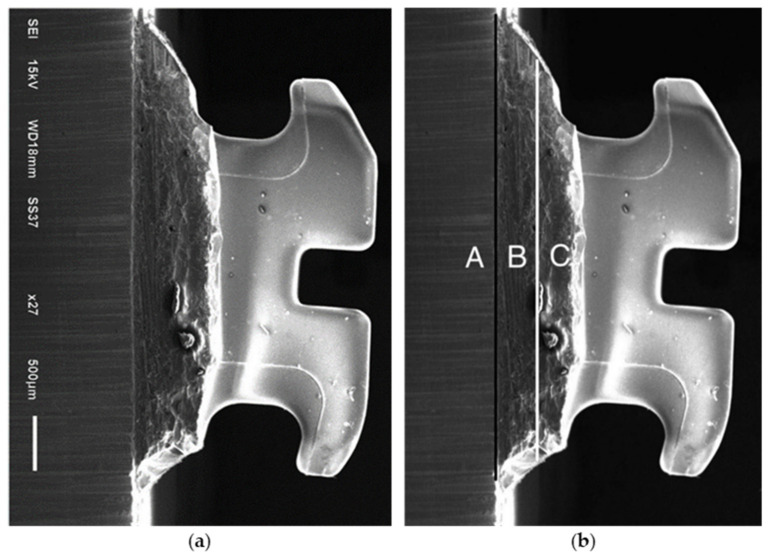
(**a**) Cross-sectional SEM image of the glazed monolithic zirconia attached to ceramic bracket (original magnification 27×, bar represents 500 μm); (**b**) A, zirconia layer; B, adhesive layer; C, bracket base layer; black line, zirconia–adhesive interface; white line, bracket base–adhesive interface.

Zirconia disks (Cercon^®^, Dentsply Sirona™, Charlotte, NC, USA), were milled into 60 small pieces (size 10 × 10 × 3 mm^2^). All of the small zirconia blocks were sintered and glazed. Sixty glazed and sintered monolithic zirconia blocks were embedded in a polyvinylchloride tube fixed with self-cured acrylic resin. All of the sample surfaces were treated with 9.6% hydrofluoric acid (Porcelain Etch, Ultradent™, South Jordan, UT, USA) for 1 min, rinsed for 30 s, and then air-dried [18]. After that, the samples were divided into 2 groups equally. Precoated ceramic and metal brackets with non-woven polypropylene fiber adhesive (3M^TM^ Clarity^TM^ Advanced Ceramic Brackets, 3M Victory Series^TM^ Bracket System, and 3M^TM^ APC^TM^ Flash-Free Adhesive, 3M ESPE, St. Paul, MN, USA) were used to attach the first and second groups, respectively. In this study, precoated orthodontic adhesive was chosen for both metal and ceramic brackets in order to avoid unequal adhesive thickness. Then, each group was divided into three subgroups based on the bonding techniques: CS: ceramic bracket with silane; CB: ceramic bracket with bonding agent; CBS: ceramic bracket with bonding agent and silane; MS: metal bracket with silane; MB: metal bracket with bonding agent; and MBS: metal bracket with bonding agent and silane (3M™ Transbond™ XT Primer and 3M™ Silane Coupling Agent, 3M ESPE, St. Paul, MN, USA). Both precoated metal and ceramic brackets were placed by using 300 g of loading force after surface preparation (Figure 3). Curing light (3M^TM^ Ortholux^TM^ Luminous Curing Light, 3M ESPE, St. Paul, MN, USA) was used to cure each distal and mesial part of the bracket for 6 s (Figure 4).

### 2.2. Data Collection

#### 2.2.1. Shear Bond Strength

A universal testing machine (Model 5566; Instron^®^ Co., Norwood, MA, USA) was used to evaluate shear bond strength. According to ISO/TS 11405:2015 (E), all samples were stored in distilled water at 37 °C for 24 h prior to the test. The Zirconia surface was parallel to the force applied, and then the vertical load was transferred to the bracket base, fabricating shear force at the bracket–zirconia interfaces. Shear bond strength was recorded at a crosshead speed of 0.75 mm/min. Then, the measured values were divided by the area of the bracket base and transformed into MPa units. Image analysis software (ImageJ software, NIH, Bethesda, MD, USA) was used to measure the base area of the bracket.

#### 2.2.2. Mode of Failure

**Figure 5:**
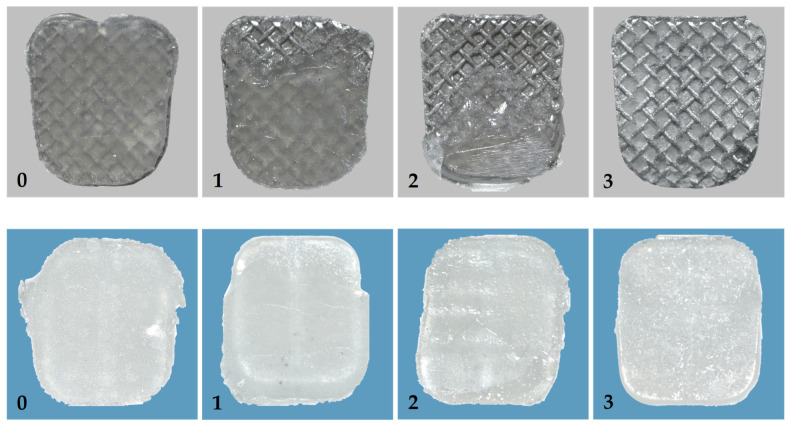
ARI, adhesive remnant index: 0, all adhesive amounts left on the bracket base; 1, ≥50% of the adhesive left on the bracket base; 2, <50% of the adhesive left on the bracket base; and 3, no adhesive left on the bracket base. The (**upper**) images show different ARI scores from metal brackets, and the (**lower**) images show different ARI scores from ceramic brackets.

After that, the brackets were examined under a 10× magnification light microscope. The micrographs were analyzed using digitized computerized image analysis software. The adhesive residual after bracket removal was evaluated considering the adhesive remnant index (ARI) and was scored based on the amount of remaining adhesive material on the bracket base; scores were expressed as a percentage of the bracket base area. The ARI was performed to categorize the sites and types of bonding failure between the zirconia surface, adhesive, and bracket. The ARI was recorded on a range of 0 to 3; 0 means all adhesive was left on the bracket base; 1 means more than half the amount of the adhesive was left on the bracket base; 2 means less than half the amount of the adhesive was left on the bracket base; and 3 means no adhesive was left on the bracket base, as shown in Figure 5 [24].

### 2.3. Data Analysis

According to the pilot study, a group of 6 samples was indicated by using G Power software (effect size = 0.8936513 and actual power = 0.9802983). The sample sizes were raised to 10 specimens per group for error compensation. 

SPSS statistical software was used to analyze the data, and descriptive statistics were used to examine the specimen characteristics. To ensure data normality, the Kolmogorov–Smirnov test was used. A one-way ANOVA was used to identify whether a significant difference existed between the shear bond strengths of various groups. For the non-homogeneity of variances, the Tamhane test was used to examine differences among multiple groups’ means. To evaluate whether a statistical test proved significant, *p* ≤ 0.05 was utilized. 

## 3. Results

### 3.1. Shear Bond Strength

**Figure 6:**
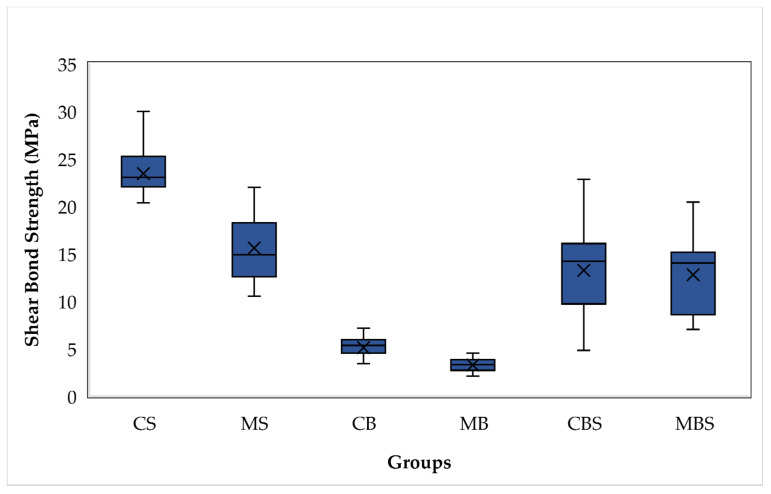
The box plots show the shear bond strength values for both ceramic and metal brackets bonded to glazed monolithic zirconia surfaces.

The descriptive statistics for the SBS values of all groups are shown in Table 1 and Figure 6. A one-way ANOVA showed that there was a statistically significant difference in mean SBS values between at least two groups (F) (5,54) = 38.683, *p* < 0.01). The Tamhane for multiple comparison tests, as in Table 1 and Table 2, demonstrated that the CS group showed significantly higher SBS values (23.42 ± 3.88 MPa) than the remaining groups. On the other hand, the MB group provided the lowest SBS values (3.26 ± 0.76 Mpa) which were not statistically different from the CB group (5.09 ± 1.50 Mpa). There was no statistical difference found among the SBS values of the MS, MBS, and CBS groups (15.57 ± 4.01, 13.23 ± 5.47, and 12.77 ± 4.43 Mpa, respectively).

### 3.2. Mode of Failure

Table 3 shows the ARI scores of the bracket bases for both the ceramic and metal brackets after debonding with a universal testing machine. The chi-square test revealed that type of bracket and attachment were related to ARI scores among six groups (chi-square = 67.422, df = 15, *p* = 0.00). The CS group showed ARI scores of 1 (70 percent), 2 (10 percent), and 3 (20 percent), showing that a significant amount of adhesive remained on the zirconia surfaces. The ARI scores of the MS, CBS, and MBS groups were mainly 1 and 2 (10/10, 10/10, and 8/10, respectively), indicating that a small amount of adhesive remained attached to the zirconia surfaces. However, the ARI scores of the samples in the CB and MB groups were all 0 (100% and 100%, respectively), which shows that there was no adhesive left on the bracket bases after the brackets broke.

## 4. Discussion

### 4.1. Shear Bond Strength

In this study, the bonding properties of ceramic and metal brackets were verified. When attached to the glazed monolithic zirconia using precoated, non-woven polypropylene fiber orthodontic adhesive, the SBS values of the CS group (ceramic brackets with silane) were much higher than those of the other groups.

According to Reynolds in 1975, the range of 5.8–7.8 MPa was the minimum SBS value required to bear regular orthodontic forces [19]. In this study, the CS, MS, CBS, and MBS groups reached the required SBS values (23.42 ± 3.88, 15.57 ± 4.01, 13.23 ± 5.47, and 12.77 ± 4.43 MPa, respectively) when bonding to glazed monolithic zirconia. However, the CB and MB groups showed lower SBS values (3.26 ± 0.76 and 5.09 ± 1.50 MPa, respectively) than the force range required for normal orthodontic tooth movement. This showed that silanization was even more important for bonding to the glazed surfaces of monolithic zirconia.

Furthermore, using only a silane coupling agent resulted in higher SBS values compared to a combination of silane and a bonding agent for both metal and ceramic brackets attached to glazed monolithic zirconia. In 2022, Charoenbhakdee et al. demonstrated that 6% (*v/v*) of silane concentration granted higher SBS values than that of 3% and 1% (*v/v*) of silane concentration when bonding composite resin to lithium disilicate glass ceramics [25]. The implication has emerged that silane concentration might be diluted when used with a bonding agent. For this reason, using silane alone provided higher SBS values in this study.

The study of the bonding properties of metal and ceramic brackets indicated that both types of brackets may offer the necessary shear bond strength for non-glazed or polished monolithic zirconia. Lee et al., in 2018, showed that Scotchbond Universal adhesive with ceramic brackets provided the highest SBS values (13.85 ± 1.48 MPa) when bonding to polished monolithic zirconia [21]. Imani et al., in 2019, reported that the sandblasting technique for surface preparation granted the highest SBS values (19.25 ± 9.07 MPa) when using metal brackets bonded to non-glazed monolithic zirconia [20]. Moreover, the study that compared metal and ceramic brackets found that metal brackets provided higher SBS values than ceramic brackets on polished monolithic zirconia for both sandblasting (23.29 ± 5.34 and 20.06 ± 4.05 Mpa) and LASER (21.59 ± 4.03 and 17.55 ± 3.88 Mpa) surface treatments [22].

On the other hand, Kwak et al., in 2016, demonstrated that hydrofluoric acid etching provided no significant difference in SBS values compared to the alumina air abrasion surface treatment (15.24 ± 3.36 and 15.78 ± 2.39 Mpa, respectively) on glazed monolithic zirconia attached to metal brackets. Furthermore, using bur roughening for surface preparation on glazed monolithic zirconia exposed the zirconia substructure. Thus, silanization for enhancing SBS values was not recommended for this method [23]. Moreover, Douara et al., in 2019, found that using hydrofluoric acid etching and silane for surface treatment on glazed monolithic zirconia provided the highest SBS values (8.15 ± 2.41 Mpa) compared to other methods [26]. Lee et al., in 2017, also stated that hydrofluoric acid and a silane coupling agent for the surface treatment of lithium disilicate improved the bond strength of resin cement [18]. In this study, the CS group, using hydrofluoric acid with only silane showed the same trend as the abovementioned studies by providing the highest SBS values compared to the others.

### 4.2. Mode of Failure

There were two types of failure modes between the two materials. First, cohesive failure arises in the bonding agent, the bracket, or even in the zirconia itself, indicating that the bond within the interfaces was tougher than the material. The second type, adhesive failure, happens at the bracket/adhesive or the zirconia/adhesive interfaces, indicating that the bond strength was even weaker at the interfaces between the adhesive and material [22]. In order to prevent the enamel structure from being damaged during bracket debonding, there should not be much adhesive remaining on the tooth surface. This also refers to restorations. A useful suggestion is to diminish cohesive failure in the zirconia surfaces and leave as little adhesive as possible on the surfaces of the zirconia. Therefore, low ARI scores and avoiding any cohesive failure within the zirconia are necessary.

In this study, pure adhesive failure at the adhesive/zirconia interface in the CB and MB groups (100 percent ARI score of 0 for both groups) indicated that there was no adhesive left on the zirconia and, therefore, no chance of damaging the zirconia surfaces. Although bracket debonding was an advantage of these bonding techniques (the CB and MB groups), the SBS values fall short of the minimal threshold for moving teeth. The adhesive was unable to adhere to the silica oxide presented in the glazed monolithic zirconia surfaces since there was no silane coupling agent used in the bonding procedure, as shown in Figure 1. Thus, the ARI scores showed only adhesive failure in these groups.

The majority of MOFs were mixed and cohesive failures inside the adhesive for the CS, MS, CBS, and MBS groups; none of these failures affected the surfaces of the brackets or zirconia. However, two samples from the CS group had an ARI score of 3, indicating that there was a huge amount of adhesive residue on the zirconia surface overall. A 1994 study found that cohesive failures in ceramic itself would occur at an excessive force of more than 13 MPa. This means that after debonding, the extremely high force of bond strength might harm the restoration surfaces [27]. This could be the only disadvantage when it comes to the debonding process. Extremely high SBS values and an excessive amount of adhesive left on the restoration could cause damage to the restorations during the removal of the bracket or adhesive. Moreover, another two samples in the MBS group showed an ARI score of 0, which was extremely satisfactory after the brackets were removed because it decreased the possibility of restoration injury.

## 5. Conclusions

The highest SBS is achieved when a ceramic bracket with a silane coupling agent is bonded to a glazed monolithic zirconia surface using precoated, non-woven polypropylene fiber orthodontic adhesive. However, silane coupling agents and bonding agents are best used to lessen the possibility of damaging the restoration during debonding and preserve a suitable shear bond strength value. Therefore, it is not recommended to bond to glazed monolithic zirconia using a bonding agent alone.

## Figures and Tables

**Table 1:** Shear bond strength (SBS) values of each group (Mpa).

Group	N	Mean ± SD	Max	Min
CS	10	23.42 ± 3.88 ^a^	29.99	16.13
MS	10	15.57 ± 4.01 ^b^	21.98	10.53
CB	10	5.09 ± 1.50 ^c^	7.15	2.12
MB	10	3.26 ± 0.76 ^c^	4.51	2.08
CBS	10	13.23 ± 5.47 ^b^	22.81	4.80
MBS	10	12.77 ± 4.43 ^b^	20.42	7.01

One-way ANOVA showed a significantly different mean shear bond strength among the groups (*p* < 0.001); ^a,b,c^ the Tamhane post hoc test showed that mean SBS values with the same superscripted letters were not significantly different (*p* > 0.05). CS, ceramic bracket with silane; CB, ceramic bracket with bonding agent; CBS, ceramic bracket with bonding agent and silane; MS, metal bracket with silane; MB, metal bracket with bonding agent; MBS, metal bracket with bonding agent and silane; SD stands for standard deviation; Min stands for minimum value; and Max stands for maximum value.

**Table 2:** Mean differences of shear bond strengths of each group (MPa).

Group	CS	MS	CB	MB	CBS	MBS
CS	-	7.85 *	18.33 *	20.16 *	10.19 *	10.65 *
MS		-	10.48 *	12.31 *	2.34	2.81
CB			-	1.83	−8.14 *	−7.67 *
MB				-	−9.97 *	−9.50 *
CBS					-	0.47
MBS						-

* The Tamhane post hoc test showed that the mean difference is significant at *p* < 0.05.

**Table 3:** The distribution of frequency and percentage of adhesive remnant index (ARI) scores of each group.

Group	N	ARI Score ^1^			
		0 (%)	1 (%)	2 (%)	3 (%)
CS	10	0 (0)	7 (70)	1 (10)	2 (20)
MS	10	0 (0)	6 (60)	4 (40)	0 (0)
CB	10	10 (100)	0 (0)	0 (0)	0 (0)
MB	10	10 (100)	0 (0)	0 (0)	0 (0)
CBS	10	0 (0)	9 (90)	1 (10)	0 (0)
MBS	10	2 (20)	6 (60)	2 (20)	0 (0)

^1^ ARI, adhesive remnant index; 0, all adhesive left on the bracket base; 1, ≥50% of the adhesive left on the bracket base; 2, <50% of the adhesive left on the bracket base; and 3, no adhesive left on the bracket base.

## Data Availability

Not applicable.
